# Comparison of Bacterial Diversity Between Two Traditional Starters and the Round-Koji-Maker Starter for Traditional Cantonese Chi-Flavor Liquor Brewing

**DOI:** 10.3389/fmicb.2018.01053

**Published:** 2018-05-23

**Authors:** Jie Wang, Qingping Zhong, Yingying Yang, Hanrong Li, Li Wang, Yigang Tong, Xiang Fang, Zhenlin Liao

**Affiliations:** ^1^College of Food Science, South China Agricultural University, Guangzhou, China; ^2^State Key Laboratory of Pathogen and Biosecurity, Beijing Institute of Microbiology and Epidemiology, Beijing, China

**Keywords:** *Xiaoqu*, bacterial diversity, MiSeq high-throughput sequencing, Round-Koji-maker starter, *Jiu Bing*, *Bing Wan*

## Abstract

*Xiaoqu* is a traditional fermentation starter that is used for Chinese liquor production. Although microorganisms in the starters are closely associated with the quality and flavor of liquor, knowledge of the microbiota in *xiaoqu* is still far from complete, let alone the starters produced by new processes. Here, Illumina MiSeq high-throughput sequencing was applied to study bacterial composition in three types of *xiaoqu* used in Cantonese soybean-flavor (*Chi*-flavor) liquor, namely two traditional starters (*Jiu Bing* and *Bing Wan*) and a Round-Koji-maker starter (*San qu*) produced by the automatic starter-making disk machine. The results showed bacterial diversity in traditional starters was similar and higher than that in the Round-Koji-maker starter. *Lactobacillus* and *Pediococcus* were the dominant genera in all starters, while other different dominant genera also existed in different starters, which were *Weissella, Acetobacter*, and *Gluconobacter* for *Jiu Bing, Weissella* for *Bing Wan*, and *Bacillus, Acetobacter, Acinetobacter* and *Klebsiella* for *San qu*, respectively. Meanwhile, *Cytophagaceae*, one particular microbial family, and some pathogens including *Klebsiella, Cronobacter*, and *Enterobacter* were also found in *San qu*, indicating the automatic starter-making disk machine should be ameliorated before applied into industrial production. These results enriched our knowledge on *xiaoqu*-related microorganisms and might be helpful in industrial *Chi*-flavor liquor production and the development of fermentation technology.

## Introduction

Many different types of liquor (*baijiu*), produced by various processes and material in different regions of China, can be distinguished based on the fermentation material, manufacturing techniques, fermentation starters, and product flavors (Zheng and Han, 2016). They were classified into 12 categories by Zheng and Han according to the final flavor of the liquor such as light flavor, strong flavor, sauce flavor, and *Chi* flavor (Zheng and Han, 2016). Soybean-flavor liquor (also called as *Chi*-flavor liquor, such as *Jiujiang* double distilled liquor and *Yubingshao* liquor), one of particular Cantonese traditional liquors in Pearl River Delta region and Southeast Asian Chinese, is named for its dense fermented soybean flavor with β-phenethyl alcohol and ethyl ester as the primary aroma compounds made from fermented soybeans, and its culture has been considered as one of the most archaic distilled alcoholic beverages over the world. The archaic liquor stems from cereals such as rice and soybean by complex fermentation processes using a natural blended cultural starter called *xiaoqu* which is one of three different types of the starters called *JiuQu* used in Chinese liquor (Gou et al., 2015), and the complex fermentation involved numerous steps, such as making starter, distilling, packing and so on. This kind of starter works as a saccharifying and fermenting agent for the production of soybean-flavor liquor and contains a variety of microorganisms, enzymes, metabolites and degradation products as well as important flavor compounds, which would contribute to the aroma of the final distillate (Gou et al., 2015).

**FIGURE 1:**
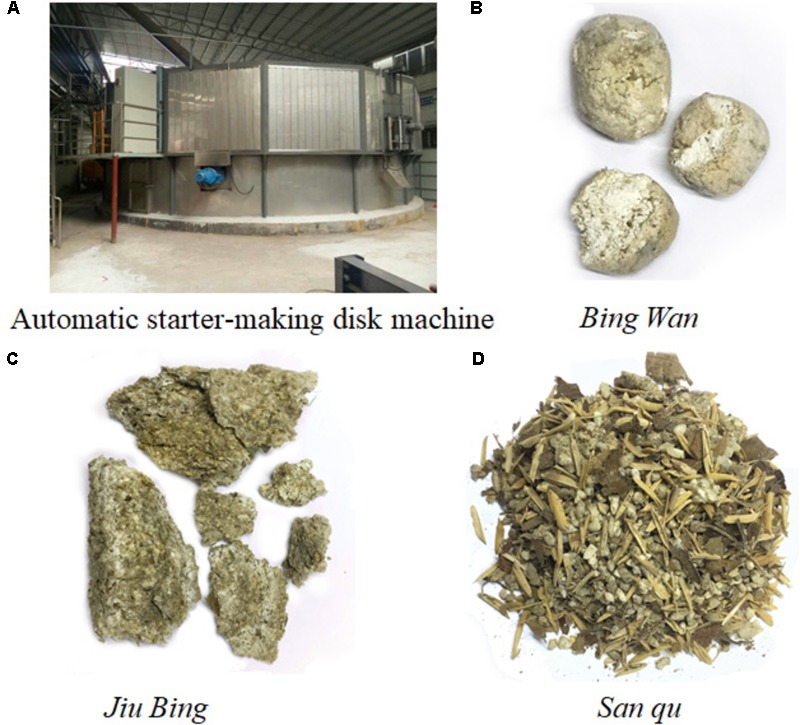
The shapes of three different types of starters and the diagram of the automatic starter-making disk machine. **(A)** Automatic starter-making disk machine. **(B)** *Bing Wan*. **(C)** *Jiu Bing*. **(D)** *San qu*.

During liquor production, the functional microbes in ***JiuQu*** contribute to the production of aromatic compounds (Liu et al., 2017). Meanwhile, the quantity and species of microorganisms such as bacteria, yeast and molds in fermented starters will be altered due to the micro-environmental changes during the fermentation process, coupled with utilization of nutrimental materials and accumulation of metabolic productions, such as pH decrease, increase of alcohol concentration, and so on (Wang H. et al., 2008). Thus, the microbial components of the starters largely determine the flavor and quality of the produced liquor. Several studies have demonstrated the diversity of the microbial community in traditional Chinese liquor *daqu*, another starter different from *xiaoqu*, indicating there are striking differences in community structure among different *daqu* samples due to the differences in grains, the fermentation time, and so on (Wang C. et al., 2008; Wang H. et al., 2008; Wang et al., 2011; Shi et al., 2009; Li et al., 2011; Liu et al., 2017; Zheng et al., 2012). Meanwhile, a few studies reported the microbial diversity in *xiaoqu*, but mainly focused on Huaxi *Xiaoqu*, Sichuan *Xiaoqu* and Hubei *Xiaoqu* (Gou et al., 2015; Wu H. et al., 2017), suggesting the bacterial community was relatively more complex than the fungal community, and different *xiaoqu* possessed different bacterial compositions. However, knowledge of the microbiota in *xiaoqu* is still far from complete, let alone a new type of starter produced by new processes, i.e., the Round-Koji-maker starter produced by using the automatic starter-making disk machine (its diagram shown in **Figure 1A**) which decreases the influence of operators and reduces labor costs and fermentation time compared with the traditional methods (Cao et al., 2017). To the best of our knowledge, the bacterial composition of a Round-Koji-maker starter named with *San qu* was analyzed and compared with that of two traditional fermentation starters named with *Bing Wan* and *Jiu Bing* by Illumina MiSeq analysis in this study.

## Materials and Methods

### Sample Collection and Preparation

*Bing Wan, Jiu Bing*, and *San qu* (their shapes shown in **Figures 1B–D**) labeled with W, B and S, respectively, were collected from Guangdong *Jiujiang* Distillery Co., Ltd., Foshan, China. *Bing Wan* (traditional), the mother starter used to produce *Jiu Bing* (traditional) and *San qu* (the Round-Roji-maker starter), is made of 100 kg of rice, 4 kg of leaves of *Isatis indigotica*, 0.3 kg of Chinese medicinal herbs, as well as 3 kg of stock culture which was produced by *Jiujiang* Distillery Co., Ltd., afterward all the materials have been shaped into a ball. *Jiu Bing* makes up of 2 kg of *Bing Wan*, 100 kg of rice, 22 kg of soybeans, and 9 kg of leaves of *Isatis indigotica*, which are later shaped into a brick before the fermentation process. *San qu*, which was fermented in Round-Koji-maker, contains the same materials as *Jiu Bing* other than some chaff and lacks the shaping process during the manufacturing procedures.

In order to obtain adequate information, three blocks of each type of starter samples (B, W, and S) were randomly selected from the upper, middle, and lower locations in the storage room, and samples B and W were promptly ground to powder while samples S remained the initial loosen condition. All samples were stored in a refrigerator at 4°C and subjected to microbial analysis within 24 h.

### DNA Extraction

Total DNA was extracted from five grams of each sample powder without chaff and leaf according to previous reports with slight modification (Schmidt et al., 1991; Li et al., 2011). Briefly, the power was suspended in 20 mL wash buffer (2% β-Mercaptoethanol, 0.2 M Tris, 0.05 M EDTA, 0.25 M NaCl, pH 8.0) with horizontal shaking at 200 rpm for 20 min at 20°C. The supernatant was discarded after centrifugation at 8000 rpm, and the pellet was dissolved in 2 mL modified extraction buffer (3% CTAB, 2% β-Mercaptoethanol, 2% PVPP,1.5 M NaCl, 100 mM Tris, 100 mM EDTA, pH 8.0) with 20 μL proteinase K (10 mg/mL) and incubated overnight at 37°C in an incubator shaker at 150 rpm. Subsequently, the whole system was mixed with 200 μL of 10% SDS and kept at 37°C for 30 min with mixing gently by inverting the tube every 5 min. The supernatants were collected after centrifugation at 8000 rpm for 20 min at room temperature and the protein and polysaccharide complexes were removed by extraction with an equal volume of chloroform-isoamyl alcohol (24:1), followed by extraction with equal volumes of phenol-chloroform-isoamyl alcohol (25:24:1). The aqueous phase was carefully collected by centrifugation at 8000 rpm for 15 min, and mixed with 0.1 volume of 3 M sodium acetate and 0.7 volume of cold isopropanol for 1 h at -20°C. The resulting nucleic acids were washed with 70% ethanol, and resuspended in sterile deinoized water.

### DNA Purification and PCR Amplification

The crude nucleic acids were purified with a glass fiber filter tube by High Pure PCR Template Preparation Kit (Roche). The concentrations and quality of purified DNA were determined by using a NanoDrop 2000 spectrophotometer (Thermo Scientific, United States) and PCR amplification, respectively. 20 ng DNA was used to amplify the bacterial 16S rRNA gene using the primers 27F (5′-AGAGTTTGATCCTGGCTCAG-3′) and 1492R (5′-GGTTACCTTGTTACGACTT-3′), and the primers 338F (5′-ACTCCTACGGGAGGCAGCA-3′) and 806R (5-GGACTACHVGGGTWTCTAAT-3′) (Anahtar et al., 2016) in 50 μL reaction mixtures with 25 μL of Phusion^®^ High-Fidelity PCR Master Mix (New England Biolabs), respectively. PCR products using the primers 27F and 1492R were used to quality testing of DNA samples. Then PCR products using the primers 338F and 806R were analyzed by 1.0% agarose gels electrophoresis and purified and quantified with an AxyPrep DNA Gel Extraction Kit (Axygen Biosciences, Union City, CA, United States) and QuantiFluor^TM^-ST (Promega) according to the manufacture’s direction, respectively.

### High-Throughput Sequencing and Sequence Analysis

Purified amplicons were pooled in equimolar volumes and sequenced on Illumina MiSeq platform (Majorbio Bioinformatics Technology Co., Ltd., Shanghai, China) using standard protocols. The raw Illumina sequences were deposited to the NCBI Sequence Read Archive^1^ with the project accession number of SAMN07336319-07336327 and quality-filtered by an open-source soft package named Quantitative Insights into Microbial Ecology (QIIME) pipeline (Caporaso et al., 2010). Operational taxonomic units (OTUs) were assigned with a 97% similarity cut-off using the UPARSE pipeline and classified taxonomically using RDP classifier against the RDP database^2^, where chimeric sequences were removed using UCHIME (Wang et al., 2007; Edgar, 2013). Rarefaction curves and the estimates of Chao1, Ace, and Simpson/Shannon, representing the species abundance, the amount of unique OTUs found in each sample, and the microbial diversity, respectively, were generated by the Mothur software (Schloss et al., 2011). Bray–Curtis distances were calculated by QIIME pipeline and the similarity and difference between the communities were depicted by Hierarchical cluster analysis and Venn diagram. Meanwhile, Principal components analysis (PCA) was performed in the Fluidigm SINGuLAR Analysis Toolset 2.0 R package, which calls the princomp R package^3^. Additionally, abundant features consistent with biologically meaningful categories were identified differentially on the OTU level by LEfSe, an algorithm for high-dimensional biomarker discovery and explanation which identifies genomic features and characterizing the differences in different conditions, on the website https://huttenhower.sph.harvard.edu/galaxy/, and the threshold on the logarithmic LDA score for discriminative features is >2.0 (Zhang et al., 2013).

#### Plate Counting

Ten grams of the samples were homogenized in 90 mL sterile phosphate buffered saline and shaken for 10 min at normal speed. Then ten twofold serial dilutions were made and 1 mL of the dilution was mixed with molten medium consisting of 5 g/L beef extract, 10 g/L peptone, 5 g/L NaCl and 15 g/L agar for pour-plating. After incubation, the colonies appearing on the plates were counted and selected randomly for cultivation in order to extract DNA and amplify the bacterial 16S rRNA gene. Obtained sequences were sequenced in Majorbio Bioinformatics Technology Co., Ltd., and blasted in NCBI website. All counts were repeated three times for each sample and results were reported as the means.

#### Volatile Acids Analysis

Volatile acids, such as formic acid, acetic acid, propionic acid, and so on, were analyzed via ion-exchange chromatography on a DIONEX ICS-3000 (Dionex Corporation, Sunnyvale, CA, United States). Briefly, the samples after fermented with three starters were filtered through Dionex OnGuard^TM^ II RP and H cartridge (Thermo, Sunnyvale, CA, United States) to remove interfering substances and was loaded into the instrument according to the instructions (Zhang and He, 2010). Volatile acids in samples were the sum of the content of each acid calculated with the standard solution.

### Data Analysis

Statistical analysis was subjected to one-factor analysis of variance performed using SPSS 18.0 (SPSS Inc., Chicago, IL, United States) and *p* < 0.05 was considered to be statistically significant differences.

## Results

### Overall Analysis of Illumina MiSeq Sequencing

**FIGURE 2:**
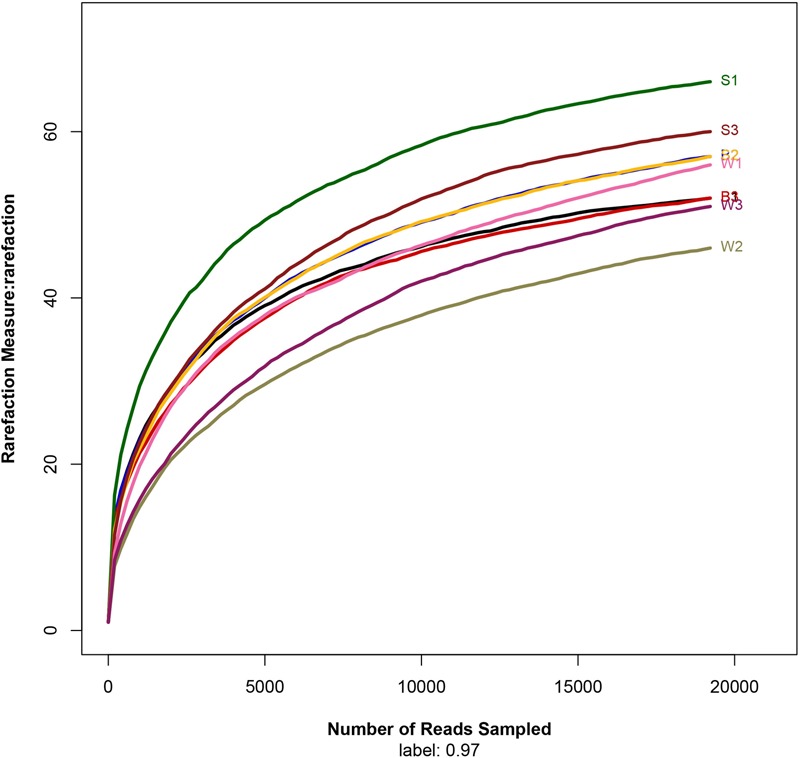
Rarefaction analysis of the different samples. Rarefaction curves of OTUs clustered at 97% sequence identity for different samples.

To assess the bacterial diversity, Illumina MiSeq sequencing was applied and a total of 19,222 valid reads and 497 OTUs were obtained from the nine samples. The samples from *Jiu Bing, Bing Wan* and *San qu* contained 52 to 57 OTUs, 46 to 56 OTUs and 57 to 66 OTUs, respectively (**Table 1**). All of the rarefaction curves based on the OTUs (**Figure 2**) tended to reach saturation, indicating that obtained sequenced reads were sufficient to represent the bacterial diversity in different samples.

**Table 1:** Microbial community richness and diversity indices of the 16S rRNA sequences for clustering at 97% sequence similarity from nine samples in three different types of starters.

Sample	OTU	Ace	Chao	Shannon	Simpson	Coverage
B1	52	57	56	1.73	0.2786	0.9996
B2	57	67	68	2.07	0.1581	0.9994
B3	52	58	58	2.07	0.1636	0.9995
S1	66	72	72	1.41	0.4768	0.9995
S2	57	67	65	1.47	0.3499	0.9994
S3	60	66	63	1.45	0.3243	0.9995
W1	56	74	76	1.69	0.2225	0.9992
W2	46	58	62	1.61	0.2351	0.9993
W3	51	63	64	1.62	0.2525	0.9993

The total number of OTUs calculated by Chao 1 estimator was 61 for *Jiu Bing* on average, 67 for *San qu* on average and 67 for *Bing Wan* on average, respectively, indicating that *Jiu Bing* had the lowest richness than the other two samples where there was no significantly difference (*p* > 0.05), which was validated by ACE indices (**Table 1**) and consistent with the differences in bacterial counts. The abundance of each species distributed in a community was assessed by Shannon diversity index which is positively correlated with Alpha-diversity and Simpson index which is negatively correlated with Alpha-diversity. The smallest Shannon index was 1.44 for *San qu* on average, followed by 1.64 for *Bing Wan* on average and 1.96 for *Jiu Bing* on average, indicating the diversity in *San qu* was lower than that in *Bing Wan* and *Jiu Bing*, which also demonstrated by Simpson indexes in **Table 1**. All samples had a high Good’s coverage (99.9%).

### Bacterial Community Profiles

**FIGURE 3:**
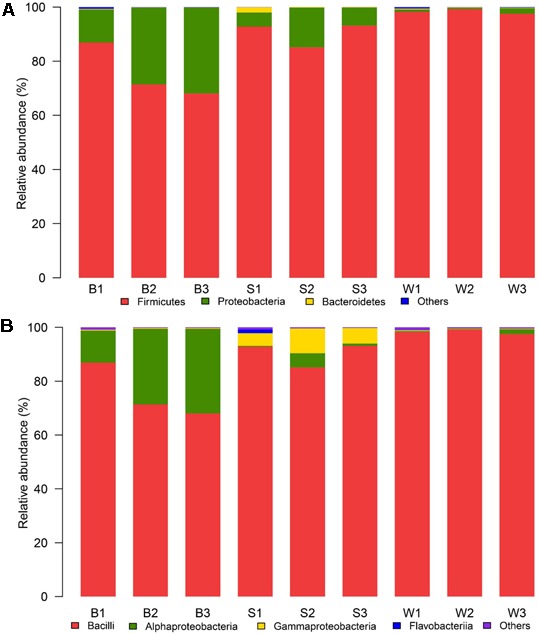
Relative abundance of the bacterial phyla **(A)** and classes **(B)** recovered from three types of starters by high-throughput Illumina sequencing. The phylum or class with relative abundance greater than 1% of the total sequences were displayed, the relative abundances less than 1% of the total sequences was defined as “Others.”

There were dissimilar bacterial communities at the phylum and class level for three types of samples (**Figure 3**). *Firmicutes* was the domain phylum in *Bing Wan, Jiu Bing* and *San qu*, accounting for 98.3, 75.4, and 90.3% of the sequences on average in each library, respectively, while *Proteobacteria*, another domain phylum in three types of samples, accounted for 24.1% of the sequences on average in *Jiu Bing*, but only for 8.9 and 1.2% in *San qu* and *Bing Wan*, respectively (**Figure 3A**). Among 18 classes detected in the nine samples, three of them were dominant (>1% abundance at least one type on average) as shown in **Figure 3B**. *Bacilli* was shared by the three types of samples with higher relative abundances than *Alphaproteobacteria* and *Gammaproteobacteria*, but *Bacilli* was the only dominant class for *Bing Wan*, and *Gammaproteobacteria* was dominant only for *San qu*.

**FIGURE 4:**
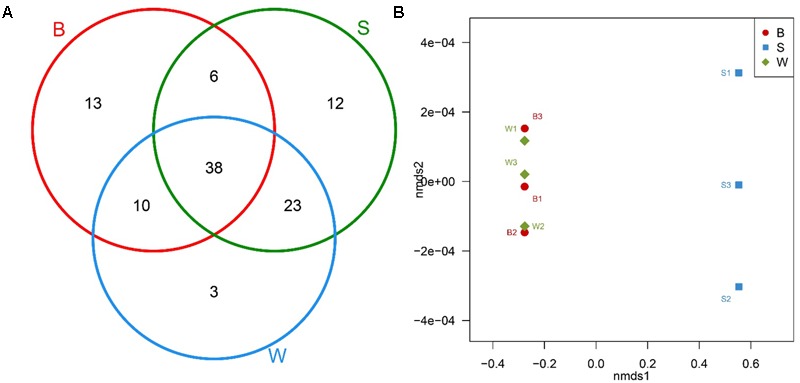
Bacterial communities comparison and shared OTU analysis across nine samples. **(A)** Venn diagram showing the unique and shared OTUs in different starters. **(B)** Non-metric multidimensional scaling (NMDS) plot generated using Bray distances between samples of three types of starters performed at the genus level.

The Venn diagram with common and unique OTUs was adopted to describe the difference and similarity among *Jiu Bing, Bing Wan* and *San qu* (**Figure 4A**). All three types of starters had a total of 105 OTUs observed and 38 of them were in common. The shared OTUs were *Firmicutes, Proteobacteria* and *Bacteroidetes*, accounting for 45.9, 37.8, and 16.2% of shared OTUs, respectively. *Bing Wan* and *San qu* had more shared OTUs (61, 58.1% of total) than any of them with *Jiu Bing* (B/S, 44, 41.9%; B/W, 48, 45.7%). The number of OTUs unique to individual starters was 13 for *Jiu Bing*, 12 for *San qu*, and 3 *Bing Wan*, respectively.

In addition, non-metric multidimensional scaling (NMDS) and hierarchical clustering analyses, which examine relationships between bacterial communities such as those of the microbial flora, were used to determine whether those OTUs identified differentiated among different types of starters. The comparisons by NMDS (genus level similarity) using the Bray–Curtis similarity metric indicated that the identified taxa significantly partitioned the starter samples into two distinct groups, with samples B clustering with samples W whereas samples S were separated by the second non-metric multi-dimensional scaling (nmds2) (**Figure 4B**). These results suggested that the new technique applied to produce *San qu* resulted in the distinction of the microbial community of the starters, and there would be similarity between *Jiu Bing* and *Bing wan* as the same traditional technique was used in their production.

### Genus-Level Distribution

**FIGURE 5:**
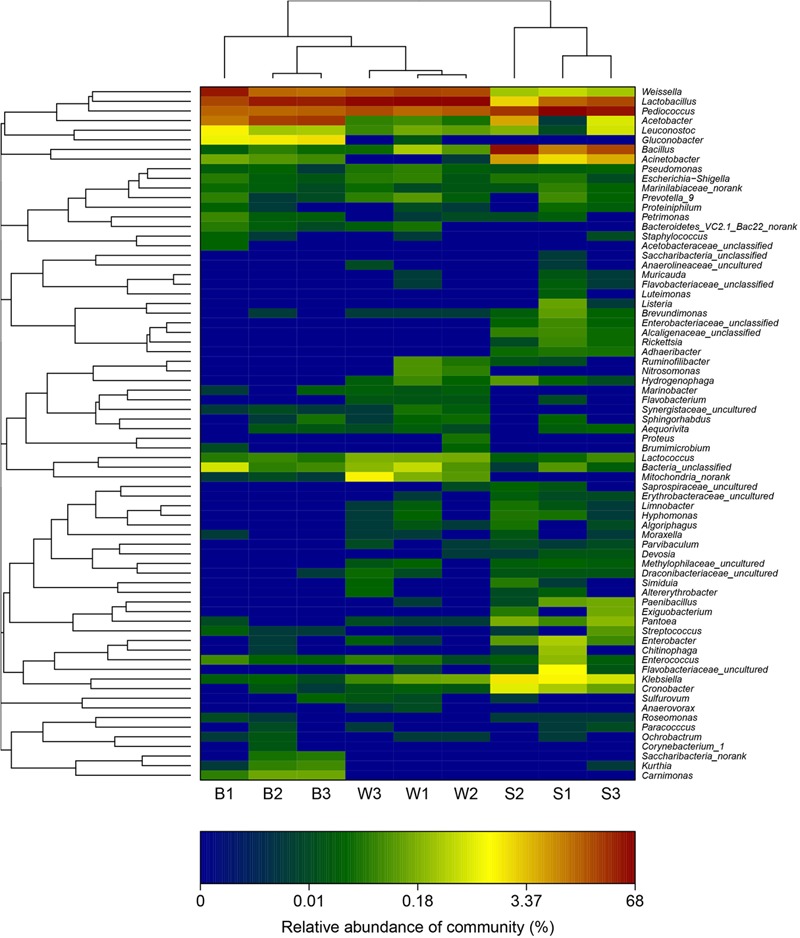
Double hierarchical dendrogram showing the bacterial distribution among nine samples. The bacterial phylogenetic tree was calculated using the approximately-maximum-likelihood method, and the relationship among samples was determined by the Bray distance and the complete clustering method. The heatmap plot depicts the relative percentage of each bacterial genus (variables clustering on the y-axis) within each sample (x-axis clustering). The relative values for bacterial genus are depicted by color intensity with the legend indicated at the bottom of the figure. Clusters based on the distance of the nine samples along the X-axis and the bacterial genera along the Y-axis are indicated in the upper and left of the figure, respectively.

**FIGURE 6:**
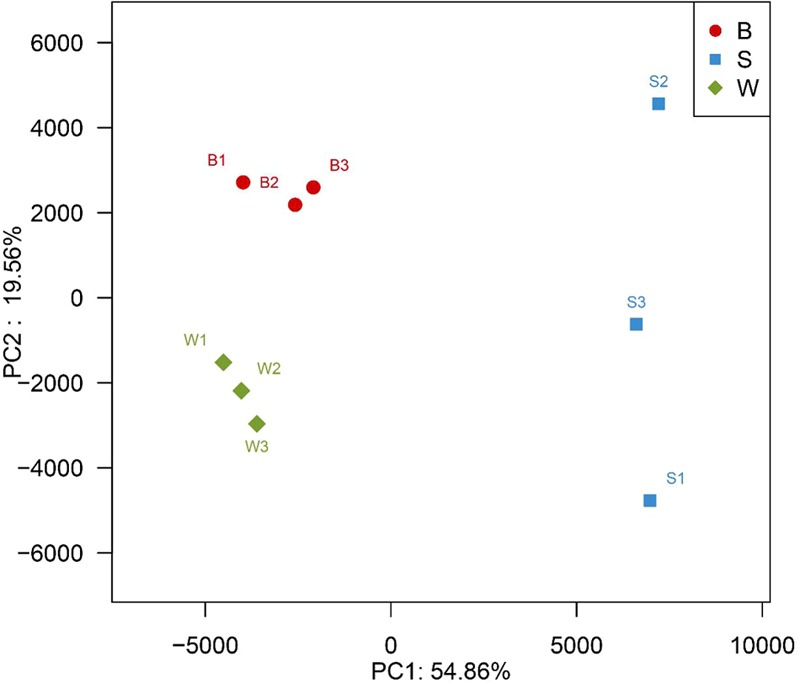
Principal component analysis of nine metagenomes from three different types of starter made by two different processes, showing the relatedness of the bacterial community in the different samples.

A hierarchically clustered heatmap analysis based on the bacterial community profiles at the genus level demonstrated that samples W grouped together at first, and then clustered with the group of sample B2 and B3, and sample B1 in order, which ultimately clustered with the group of samples S where the sample S1 clustered with sample S3 and S2 in order (**Figure 5**), indicating the distinction of bacterial genus in samples S significantly differed from those in samples B and W, which was consistent with the results obtained from NMDS and hierarchical clustering analyses. There were a total of seventy-two taxa observed in three types of starters. For samples S there were six dominant genera including *Lactobacillus, Pediococcus, Bacillus, Acetobacter, Acinetobacter*, and *Klebsiella*, while three dominant genera (*Lactobacillus, Pediococcus*, and *Weissella*) exited in both samples B and W, but samples B harbored two other dominant genera (*Acetobacter* and *Gluconobacter*) which accounted for a small proportion in samples W. Specifically, the relative abundance of four dominant genera including *Pediococcus* (48.7% on average), *Bacillus* (28.3% on average), *Acinetobacter* (4.3% on average) and *Klebsiella* (1.4% on average) in samples S was clearly higher than that in samples B and W, and seven special genera including *Cronobacter, Enterobacter, Flavobacteriaceae, Klebsiella, Paenibacillus, Pantoea*, and *Rickettsia* existed in samples S but not or few in samples B or W, while the relative abundance of *Weissella* and *Lactobacillus* in samples S was apparently lower than those in samples B and W, and the absence of *Synergistaceae* was apparent in samples S. Compared with samples W, samples B possessed lower abundance of *Lactobacillus* (36.0% on average), one of three shared dominant genera in samples B and W and *Leuconostoc*, but higher abundance of *Acetobacter* (22.1% on average), *Gluconobacter* (1.7% on average), and *Acinetobacter* (0.1% on average). Moreover, *Carnimonas* and *Kurthia* are peculiar to samples B. Meanwhile, principal component analysis (PCA) was performed to reveal the relationships among the different samples (**Figure 6**). On the right side of the graph along the first principal component axis (PC1) accounting for 54.86% of the total variations, were samples S which were gathered together. Although samples B were close to samples W, they were distinctly separated by the second component (PC2) which accounted for 19.56% of the total variation. Overall, the PC1 and PC2 axes explained 74.42% of the variations between the differences of the bacterial communities. These data indicated that three types of starters had different characteristic bacterial communities, which was in agreement with heatmap analysis.

**FIGURE 7:**
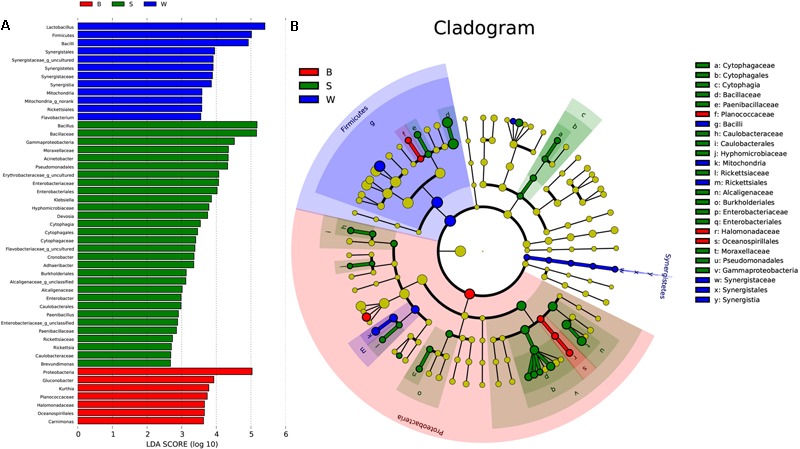
Key phylo-types of bacteria analysis in three types of starters by using LEfSe. **(A)** Indicator bacterial groups with the three types of starters with LDA values higher than 2.0. **(B)** Cladogram indicating the phylogenetic distribution of microbial species associated with the three types of starter. Species with LDA values of 2.0 or higher determined by LEfSe are displayed. Differences are represented in the color of the most abundant class. The diameter of each circle is proportional to the abundance of the taxon. The strategy of multiclass analysis is nonstrict (at least one class differential). Circles represent phylogenetic levels from domain to genus inside out. Labels are shown of the phylum, class, and family levels.

Linear discriminate analysis (LDA) effect size (LEfSe) was used to find biomarkers from metagenome data to identify potential distinguishable taxa in three types of starters. A total of 181 bacterial groups were well-defined to at least one type of starter using the default logarithmic (LDA) value of 2.0, and Cladograms showed taxa with LDA values higher than 2.0 for lucidity (**Figures 7A,B**). We detected that seven groups of bacteria were enriched in *Jiu Bing* (B), namely, *Proteobacteria, Gluconobacter, Kurthia, Planococcaceae, Halomonadaceae, Oceanospirillales*, and *Carnimonas*. Within these, one conspicuous species, *Proteobacteria*, had an LDA value higher than five (**Figure 7A**). In *San qu* (S), the major bacterial families were *Cytophagaceae, Moraxellaceae, Enterobacteriaceae, Alcaligenaceae, Rickettsiaceae, Hyphomicrobiaceae, Caulobacteraceae, Paenibacillaceae*, and *Bacillaceae* (**Figure 7B**), and Only *Bacillaceae* and *Pseudomonadales* showed LDA values higher than five (**Figure 7A**). Meanwhile, the *Firmicutes* phylum and *Synergistia* phylum were enriched, particularly the *Bacilli* and *Synergistales*, respectively, and another group, *Rickettsiales*, was also rich in *Bing Wan* (W) (**Figure 7B**). At precise taxonomy levels, *Bing Wan* only had *Lactobacillus* and *Firmicutes* showing LDA values higher than five (**Figure 7A**).

Overall, two phyla, two classes, five orders, nine families, and two genera were significantly higher in *San qu*, compared with other two starters (**Figure 7B**). *Cytophagia* and *Gammaproteobacteria* were enriched from class to genus level, while *Caulobacteriales* and *Burkholderiales* were enriched from order to family level. In contrast, the enrichment of *Firmicutes* and *Synergistetes* could be observed in *Bing wan*. Moreover, members from *Proteobacteria* were enriched in *Jiu Bing*. Notably, at the class level, microbes from *Cytophagia* were unique in *San qu*, and those from *Synergistetes* were only determined in *Bing Wan*, with those from *Oceanospirillales* significantly more prominent in *Jiu Bing*. More specifically, the overrepresented genera in *San qu* included *Pediococcus, Bacillus, Acinetobacter, Klebsiella*, and *Cronobacter*, and in particular *Adhaeribacter*, was also detected as the unique microbe. On the other hand, *Lactobacillus, Weissella*, and *Pediococcus* were found in all three types of the starters despite the level of *Weissella* in *San qu* being significantly lower than those in the other two starters, while *Acetobacter* and *Gluconobacter* were distinctly abundant in *Jiu Bing* but not in *Bing Wan*.

### Plate Count, Isolated Bacteria and Volatile Acid Analysis

Bacterial counts were detected by plating serial dilutions of samples, and the number was 41.5 × 10^6^ on average for *Bing Wan*, 1.56 × 10^6^ on average for *Jiu Bing*, and 11 × 10^6^ on average for *San qu*, respectively, indicating the bacterial diversity and/or compositions may be quite different among three starters. Moreover, a total of 20 bacterial strains in each of three starters were randomly selected and identified by sequencing the 16S rRNA. *Bacillus tequilensis, Bacillus amyloliquefaciens*, and *Pediococcus lolii* were found and accounted for 66.67, 16.67, and 16.67%, respectively, in *San qu*. *Lactobacillus pentosus* and *Undibacterium oligocarboniphilum* were found in *Jiu Bing*, accounting for 33.33 and 66.67%, respectively. However, only *Pediococcus pentosaceus* was encountered in *Bing Wan*. The obtained results testified that *Lactobacillus, Bacillus, Pediococcus*, and *Undibacterium* (belonging to *Proteobacteria* phylum) are the most dominant genera for *Bing Wan, San qu*, and *Jiu Bing*, respectively, which coincided with the heatmap (**Figure 5**) and the LDA scores (**Figure 7A**). Furthermore, volatile acids after fermentation using three starters were measured by ion chromatography and the contents were 0.23 g/L on average for *Bing Wan*, 0.52 g/L on average for *Jiu Bing*, and 0.89 g/L on average for *San qu*, respectively, which might be due to the different bacterial counts and/or compositions among three starters.

## Discussion

The present study is the first report to compare the bacterial diversity between the traditional starters and the Round-Koji-maker starter. The results indicated the traditional starters (*Jiu Bing* and *Bing Wan*) showed higher bacterial richness and diversity than that in the Round-Koji-maker starter (*San qu*), and bacterial composition in the three *xiaoqu* starters mainly included *Lactobacillus, Pediococcus, Weissella, Bacillus, Acetobacter, Acinetobacter, Gluconobacter*, which was different from that in Huaxi *xiaoqu*, Sichuan *xiaoqu* and Hubei *xiaoqu*, where the dominant bacteria are *Weissella, Staphylococcus, Empedobacter*, and *Corynebacterium* for Huaxi *xiaoqu, Weissella, Staphylococcus, Corynebacterium, Bacillus*, and *Leuconostoc* for Sichuan *xiaoqu*, and *Weissella, Empedobacter, Sphingobacterium, Acinetobacter, Bacillus, Pseudomonas*, and *Flavobacterium* for Hubei *xiaoqu*, respectively (Gou et al., 2015; Wu H. et al., 2017). Meanwhile, slight differences existed in three samples of *San qu* (**Figures 4B, 6**), which might be caused by instability of quality during storage. The bacterial composition in tested *xiaoqu* starters was also significantly different from that in *daqu* where different samples consisted of different bacteria, for example, the dominant bacterial species were *Bacillus, Virgibacillus, Lactobacillus*, and *Trichococcus* in *Baiyunbian* liquor fermentation starters, *Bacillus, Thermoactinomyces, Staphylococcus, Lactobacillus, Micrococcineae, Brachybacterium, Enterobacter, Peniococcus*, and *Brevibacterium* in the starter of *Fen* Liquor, and *Bacillus, Acetobacter, Lactobacillus, Clostridium* in the starter of *Maotai*-flavor liquor, respectively (Wang C. et al., 2008; Wang H. et al., 2008; Shi et al., 2009; Zheng et al., 2012; Xiong et al., 2014). The dominant genera detected in all three types of starters were *Pediococcus, Lactobacillus*, and *Weissella*, all of which are main lactic acid bacteria (LAB) typically found in fermenting matter (Nout, 2009), and which were also separated from the alcoholic fermentation stage of *Shanxi* aged vinegar (Wu et al., 2012). Lactic acid was identified as a precursor of ethyl lactate, contributing to the major flavor compounds in strong aromatic liquor and affecting both the technological properties and microbial stability of the final products (Yousif et al., 2010; Wang et al., 2011; Ding et al., 2014). Also, as they can yield lactic acid from glucose or starch and provide substrates for esterification of yeasts, LAB cannot be absent from Chinese liquor (Li et al., 2011). It was also pointed out that lactic acid is able to further react with ethanol to yield the ethyl lactate (Ding et al., 2015), which constitutes the major flavor components of *Jiujiang* soybean-flavor liquor (Zhang and He, 2010). However, compared with two traditional starters, *Lactobacillus* was significantly lower and *Weissella* was even absent in the Round-Koji-maker starter (*San qu*), which resulted in both lactic acid ethyl ester and ethyl ester are noticeably lower in liquor produced by Round-Koji-maker starter (data not shown). Moreover, *Bacillus* became one of major genera in *San qu*, but was extremely sparse in both *Jiu Bing* and *Bing Wan*, which may be due to the richness of *Lactobacillus* that might synthesize antibiotics and bacteriocins resulting in growth suppression of *Bacillus* in the two traditional starters (Pal et al., 2010), and the competition and consumption of nutrition between two genera in both traditional starters, as well as the non-compact, i.e., loose, condition allowing the Round-Koji-maker starter to be exposed to air which provided *Bacillus* with sufficient oxygen for the robust proliferation. Compared with the two traditional starters, the richness of *Bacillus* in *San qu* may lead to the increase of volatile acids, which are the synthetic precursors of esters, because *Bacillus* was the important source of proteases and amylases and resulted in a precursor-rich environment which is useful for subsequent reactions leading to flavor production (Pal et al., 2010). which might cover the shortage of lactic acid ethyl ester and ethyl ester in *San qu* and so the flavor components of final liquor products using all three types of starters to ferment are not significantly different according to the information provided by *Jiujiang* Distillery Co., Ltd. In conclusion, the relatively low abundance of *Lactobacillus* and *Weissella* and high abundance of *Pediococcus* and *Bacillus* can potentially be used to distinguish the Round-Koji-maker starter from the traditional starters.

*Acetobacteraceae*, ubiquitous in nature on various plants such as fruits, cereals and herbs, can oxidize many types of sugars and alcohols to organic acids as final products during the fermentation process with an obligate aerobic metabolism (Sengun and Karabiyikli, 2011). It was reported that acetic acid is abundant in the strong-flavor liquors and could contribute positively to the flavor by yielding esters such as isoamyl acetate and 2-phenylethyl acetate (Sun et al., 2016). Here, a great abundance of acetic acid bacteria (AAB), *Acetobacter*, and *Gluconobacter*, appeared only in *Jiu Bing* among all types of starters while *Gluconobacter* was absent in *San qu*.

In addition to *Bacillus* and LAB genera, other bacteria such as *Enterobacter, Klebsiella, Cronobacter*, and *Acinetobacter* were also detected and occupied a noticeable position in the bacterial community in Round-Koji-maker starter but were rarely detected in the traditional starters, which may be attributed to the loose states caused by the production process lacking shaping of koji, which provided these microbes with suitable conditions for incubation. On the other hand, the difference in the abundance of *Cronobacter* might result from the raw ingredients, such as the grain chaff, which may be a vital source of *Cronobacter* in the food chain (Baumgartner et al., 2009). Pathogenicity of these bacteria is widely known and most of them are regarded as food-borne bacterial pathogens, but some pathogens could actually participate in flavor formation, for instance, *Enterobacter* can produce amylases and lipases (Li et al., 2015). As the existence of these food-borne bacterial pathogens, the mechanically prepared Round-Koji-maker starter could not be applied directly into industrial liquor production like the mechanically prepared *daqu* (Xiong et al., 2014) and the present technique should be improved in the future.

The most unique bacteria in the Round-Koji-maker starter samples was *Cytophagaceae*, whose members are widely distributed in nature and could be isolated from aquatic, terrestrial, and air samples but also represent Gram-negative chemoorganotrophic with primarily respiratory metabolism. Previous studies suggested that many members of *Cytophagacea* played a crucial role in digesting macromolecules such as proteins and polysaccharides (McBride et al., 2014), so we inferred that the presence of *Cytophagaceae* in the Round-Koji-maker starter may be beneficial to utilize the grain chaff by digestion of the coarse fiber of the chaff. Also, the certain *Adhaeribacter*, which was previously isolated from forest soil (Zhang et al., 2009), was found in a small amount in *San qu*, but its effects in liquor production were still unknown, which would be further investigated. So far, the *San qu* has not been used in actual production process including but not limited to the reasons mentioned above, but *Jiu Bing* and *Bing wan* are used as starter for making of Jiujiang Double Distilled and Jiujiang Twelve Square (Wu Z. et al., 2017), two traditional Chinese Cantonese soybean-flavor liquor (Cantonese rice liquor) for more than six decades.

## Conclusion

This is probably the first study to compare the bacterial diversity between the traditional starters and the Round-Koji-maker starter by using Illumina MiSeq sequencing platforms. The main bacteria were *Lactobacillus, Pediococcus, Weissella, Bacillus, Acetobacter* in three different types of *xiaoqu*, but different types of starters harbored different and specific microbial communities and abundances, which might be due to the differences in the raw materials and the conditions of their incubation. On the other hand, the Round-Koji-maker starter contained a high proportion of sample-specific bacteria which were regarded as candidate biomarkers to distinguish from traditional starters. Furthermore, some pathogens were also detected in the Round-Koji-maker starter, which suggested that the automatic starter-making disk machine should be ameliorated from the perspective of the bacterial results before being applied into industrial liquor production. Accordingly, our findings provide useful information for liquor production and process improvement based on *xiaoqu* fermentation. Additionally, further investigation will be needed to have a deeper insight into *xiaoqu*, as well as the Round-Koji-maker starter regarding other aspects, such as the fungal communities, and the development of microbes in the *xiaoqu* preparation and ripening process as well as production in different regions using different culture conditions.

## Author Contributions

JW analyzed the data and prepared the manuscript. QZ contributed to manuscript discussion. YY and HL designed and performed the experiments. LW and YT contributed to manuscript revision. XF and ZL contributed to the experimental design, manuscript revision, and overall support of this study.

## Conflict of Interest Statement

The authors declare that the research was conducted in the absence of any commercial or financial relationships that could be construed as a potential conflict of interest.

## Footnotes

1 http://www.ncbi.nlm.nih.gov/Traces/sra

2 http://rdp.cme.msu.edu/

3 http://stat.ethz.ch/R-manual/R-patched/library/stats/html/princomp.html
